# Generation of 3D retinal tissue from human pluripotent stem cells using a directed small molecule-based serum-free microwell platform

**DOI:** 10.1038/s41598-022-10540-1

**Published:** 2022-04-22

**Authors:** Hassan Rashidi, Yeh Chwan Leong, Kerrie Venner, Hema Pramod, Qi-Zhen Fei, Owen J. R. Jones, Dale Moulding, Jane C. Sowden

**Affiliations:** 1 Stem Cells and Regenerative Medicine Section, Birth Defects Research Centre, UCL Great Ormond Street Institute of Child Health, University College London and NIHR Great Ormond Street Hospital Biomedical Research Centre, 30 Guilford Street, London, WC1N 1EH UK; 2 UCL Institute of Neurology, Queens Square, University College London, London, UK

**Keywords:** Stem cells, Stem-cell differentiation, Retinal diseases

## Abstract

Retinal degenerative diseases are a leading cause of blindness worldwide with debilitating life-long consequences for the affected individuals. Cell therapy is considered a potential future clinical intervention to restore and preserve sight by replacing lost photoreceptors and/or retinal pigment epithelium. Development of protocols to generate retinal tissue from human pluripotent stem cells (hPSC), reliably and at scale, can provide a platform to generate photoreceptors for cell therapy and to model retinal disease in vitro. Here, we describe an improved differentiation platform to generate retinal organoids from hPSC at scale and free from time-consuming manual microdissection steps. The scale up was achieved using an agarose mould platform enabling generation of uniform self-assembled 3D spheres from dissociated hPSC in microwells. Subsequent retinal differentiation was efficiently achieved via a stepwise differentiation protocol using a number of small molecules. To facilitate clinical translation, xeno-free approaches were developed by substituting Matrigel™ and foetal bovine serum with recombinant laminin and human platelet lysate, respectively. Generated retinal organoids exhibited important features reminiscent of retinal tissue including correct site-specific localisation of proteins involved in phototransduction.

## Introduction

Retinal degenerative diseases are a leading cause of irreversible blindness, which have few effective treatments to date. The underlying causes of outer retinal degeneration vary and consist of a wide range of genetic and environmental factors, which result in gradual deterioration of photoreceptor cells and/or retinal pigment epithelium (RPE)^1,2^. Due to the lack of spontaneous regeneration in the human retina, cell replacement therapy is considered as a promising clinical intervention following degeneration of retinal cells^3,4^. Transplantation of RPE cells as a treatment for age related macular degeneration is already at the stage of clinical trials in a number of centres^5,6^. Pre-clinical studies of photoreceptor cell transplantation have demonstrated improved visual function in mouse inherited retinal disease models^7–13^; however, access to human photoreceptors at scale for transplantation remains a challenge.

By virtue of their unlimited self-renewal capacity and ability to differentiate into most cell types found in the human body, hPSCs are considered a suitable source for the generation of human photoreceptor cells for transplantation. Early methodologies using conventional adherent two-dimensional (2D) culture^14,15^ which showed limited retinal cell differentiation, have been superseded by three-dimensional (3D) retinal organoid platforms^16–19^ and hybrid 2D/3D^20^ and 3D/2D/3D approaches^21–24^. These retinal organoid cell culture systems self-organise to form neuroepithelial structures that resemble embryonic optic vesicles (OVs), and then mimic the cell interactions that occur during retinogenesis to produce 3D neural retinal tissue that includes an outer layer of photoreceptor cells^25,26^.

Published protocols are however labour intensive and time consuming as they often rely on multi-step approaches to form and then manually micro-dissect the OVs. In hybrid 2D/3D protocols, OVs form spontaneously from confluent 2D cultured hPSC in a period over 6–8 weeks before being manually micro-dissected and cultured individually or in small numbers in suspension^20,27^. Alternatively, an initial 3D embryoid body (EB) step is followed by retinal differentiation and microdissection of OVs budding from floating spheres^17^. In some other protocols, an initial 3D embryoid body (EB) step and spontaneous differentiation followed by 2D culture in Matrigel (MG)-coated plate have been used to form and isolate OVs by micro-dissection^21,22,24,26^.

While OV differentiation using these latter 3D platforms generates photoreceptor cells organised in a presumptive outer nuclear layer (ONL) and expressing genes involved in phototransduction, hPSC-derived photoreceptors are yet to make a therapeutic impact. Translation into the clinic is hindered, in part, by the reliance of current protocols on animal-derived supplements, low efficiency, low yield and the requirement for manual microdissection to isolate 3D OV-like structures during the differentiation protocols.

In this study, we sought to overcome some of these limitations. We initially developed a modification of a 2D/3D protocol^20,27^ by utilising small molecules at various stages of retinal differentiation. We then modified the stepwise protocol by replacing Matrigel™ (MG) (derived from mouse tumour cells) and foetal bovine serum (FBS) with recombinant laminin isoform 521 (rLN-521) and human platelet lysate (HPL), respectively, to develop a 2D/3D serum-free approach. Finally, we established a small molecule microwell-based 3D platform to circumvent the cumbersome manual OV microdissection step enabling the generation of retinal organoids at scale under both FBS-supplemented and serum-free conditions.

## Results

### Small molecule directed 2D/3D retinal differentiation

**Figure 1:**
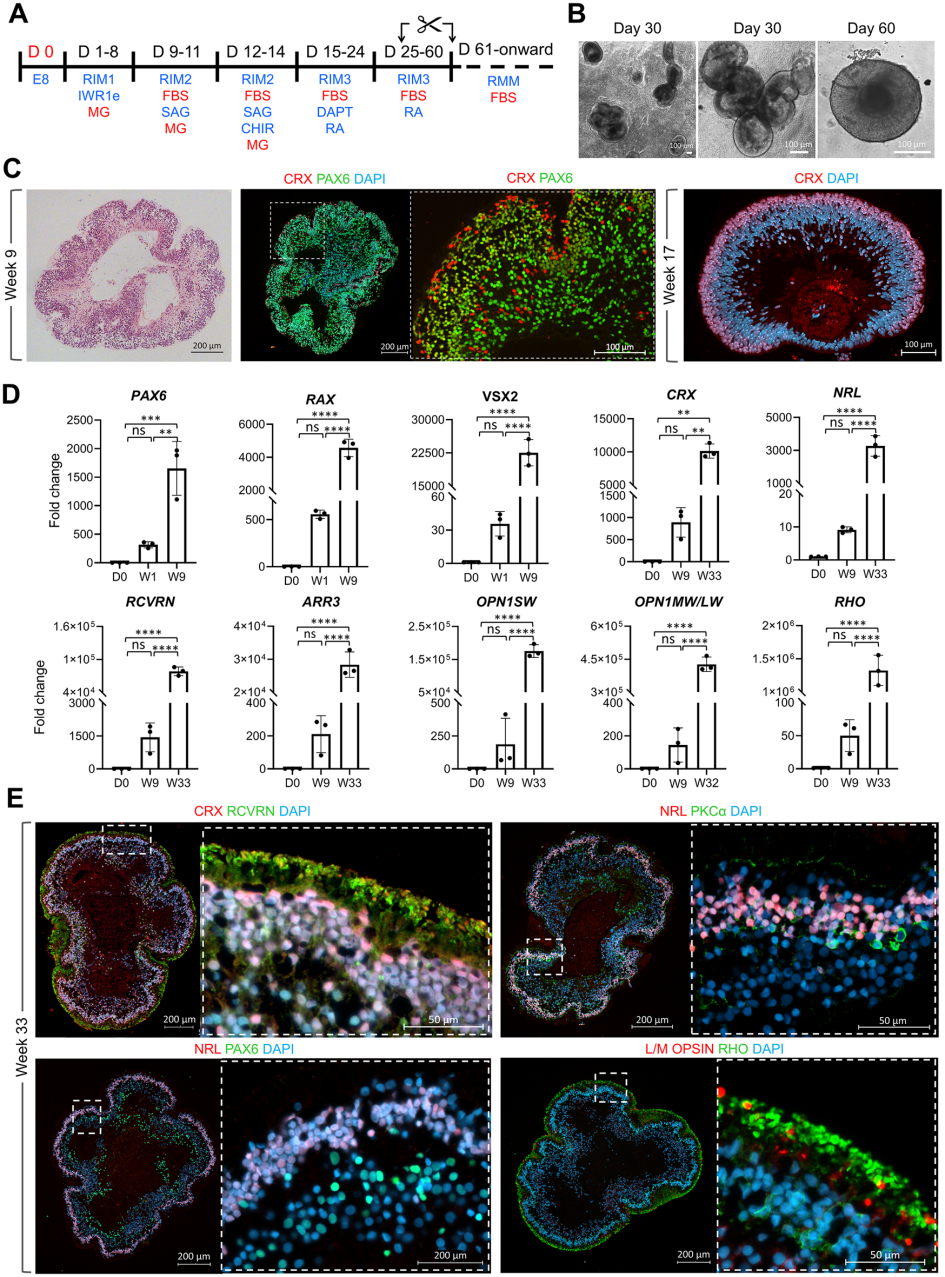
2D/3D small molecule-based differentiation of hPSC into retinal organoids using animal-derived supplements (xeno). (**A**) Schematic of the 2D/3D xeno small molecule protocol (**B**) Bright-field microscopy of 3D optic vesicle-like structures at day 30 and manually picked organoid at day 60 of differentiation (MShef10; scale bars 100 µm) (**C**) Immunohistochemistry analysis of retinal organoids at week 9 and 17 of differentiation, (MShef4 and MShef10 ESC lines respectively). (**D**) RT PCR analysis of relative expression of retinal marker genes (mean ± SD; n = 3 independently cultured sets of 10 OVs from N = 1 ESC line, MShef4). (**E**) Immunohistochemistry analysis of retinal markers at later week 33 stage of differentiation (MShef4 ESC line). ONL, presumptive outer nuclear layer in 3D organoid.

2D/3D protocols that rely on spontaneous differentiation and self-organisation of confluent 2D-cultured hPSCs^20^ achieve successful formation of OVs and subsequent differentiation into photoreceptors, but the yield is varied and cell line-dependant. To improve the efficiency and reproducibility, we developed a modified protocol by adapting a regimen of small molecules, which was previously used for differentiation of hPSC into 3D retinal organoids^17^. While we maintained the sequence of the small molecules, the times of exposure were optimised to be compatible with a 2D configuration (Fig. 1A). Similar to the Nakano protocol, cell specification was induced initially by inhibition of the WNT pathway using IWR1e, followed by stimulation of sonic hedgehog and WNT and finally inhibition of NOTCH signalling by DAPT. In addition, 1% MG was added as a supplement to the culture media in the first 14 days of differentiation to promote self-organisation and formation of retinal OV-like structures. Using this approach, OVs gradually start to form in culture from day 20 of differentiation and can be manually micro-dissected from day 30 of differentiation onward and cultured in suspension in medium supplemented with FBS, retinoic acid (RA) and taurine. Formation of OVs was consistently observed in 3 tested embryonic stem cell (ESC) lines; Fig. 1B shows representative examples from mShef10 ESC line. The yield varied from > 40 OVs developing per well of a 6-well plate in two ESC lines (mShef10 and mShef4) to < 10 in a third ESC line (mShef12), with 90% ± 5 of micro-dissected organoids forming OVs. Figure 1C shows representative examples of Haematoxylin and Eosin staining (H&E) and immunohistochemistry (IHC) of MShef4 and MShef10 OVs. Neuroectoderm marker paired box protein 6 (PAX6) and photoreceptor differentiation marker cone-rod homeobox (CRX), were detected by IHC in OVs from week 9 of differentiation confirming retinal specification (Fig. 1C). RT-PCR analysis confirmed the early upregulation of eye field-specific markers such as *PAX6* and *RAX* at week 1 of differentiation, followed by expression of retinal progenitor cell marker *VSX2* at week 9 of differentiation (Fig. 1D). Upregulation of photoreceptor-specific genes including pan photoreceptor markers *CRX*, RECOVERIN (*RCVRN*), rod-specific transcription factor neural retina leucine zipper *NRL*, cone arrestin *ARR3*, cone opsins *OPN1SW* and *OPN1MW/LW* and rhodopsin *RHO* was observed at later stages of differentiation (Fig. 1D). IHC showed that after a longer period of differentiation, the retinal neuroepithelia became more organised and developing cone and rod photoreceptor cells in the presumptive outer nuclear layer of the organoid were labelled with CRX and RCVRN, and either NRL, RHO or L/M opsin (Fig. 1E). Bipolar cell marker, PKCα labelled cells in the presumptive inner nuclear layer basal to the NRL + ve rod photoreceptors (Fig. 1E). From week 17 onward, segment-like (OS) structures protruded from the outermost nuclear layer. Phototransduction proteins such as RHODOPSIN (RHO) and long-wave sensitive opsin (L/M-OPSIN) could be detected in the segment-like (OS) structures (Fig. 1E; Supplementary Fig. 1A). Outer and inner plexiform-like layers were distinguished by labelling with synaptic marker synaptophysin (Supplementary Fig. 1A); ZO1 labelled tight junctions at the outer limiting membrane confirming the outer apical polarity of the retinal neuroepithelium with RHO-labelled segments extended outside of the OLM (Supplementary Fig. 1A).

### Development of xeno-free small molecule directed 2D/3D retinal differentiation

**Figure 2:**
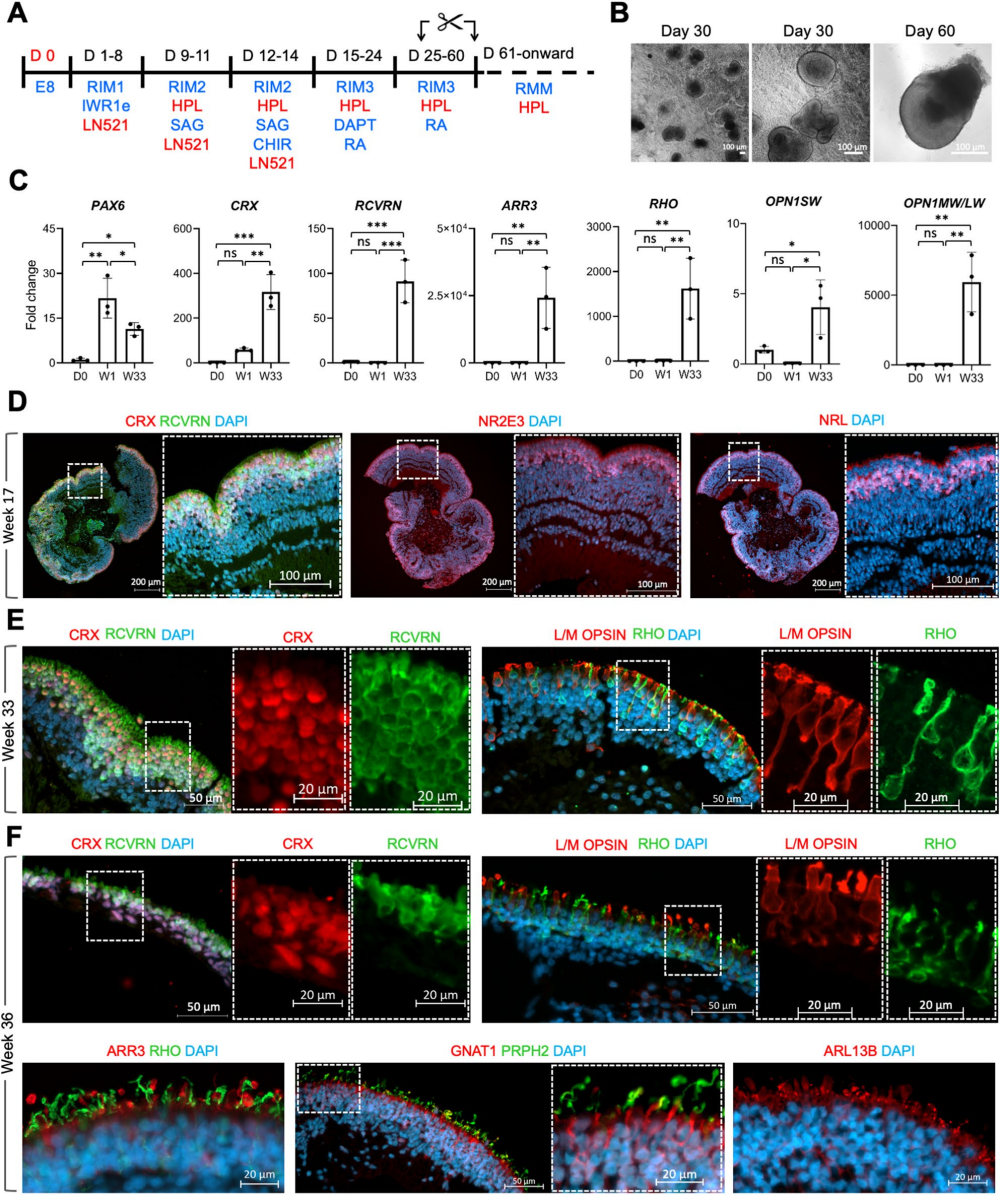
Small molecule-based 2D/3D differentiation of hPSC into retinal organoids under xeno-free condition. (**A**) Schematic of the 2D/3D xeno-free small molecule protocol (**B**) Bright-field microscopy of 3D optic vesicle-like structure at day 30 and manually picked organoid at day 60 of differentiation (scale bars 100 µm) (**C**) RT PCR analysis of relative expression of retinal marker genes (mean ± SD; n = 3 independently cultured sets of 10 OVs from N = 1 ESC line, MShef10). (**D**) Immunohistological analysis of retinal organoids at week 17 of differentiation (generated from MShef10 ESC line; scale bars 100 µm). (**E**) Immunohistological analysis of CRX, RCVRN, OPN1MW/LW, and RHO in retinal organoids at week 33 of differentiation (generated from MShef10 ESC line). (**F**) Immunohistological analysis of CRX, RCVRN, OPN1MW/LW, ARR3, RHO, GNAT, PRPH2, ARL13B in retinal organoids (from MShef10 ESC line) at week 36 of differentiation.

To facilitate clinical translation of hPSC-derived photoreceptors, we evaluated the feasibility of MG and FBS substitution with rLN521 and HPL, respectively in the small molecule directed 2D/3D protocol (Fig. 2A,B; referred to as xeno-free 2D/3D). Similar to OVs generated using animal-derived supplements, upregulation of *PAX6* as an early marker of eye-field development was observed at week 1 of differentiation (Fig. 2C). Upregulation of photoreceptor-specific genes including *CRX*, *RCVRN*, *ARR3*, *RHO*, *OPN1SW* and *OPN1MW/LW* was observed in micro-dissected OVs at later stages of differentiation indicating formation of rod and cones under serum-free conditions (Fig. 2C). IHC analysis showed that generated OVs formed self-organised retinal neuroepithelium with apically located photoreceptors labelled with CRX, NR2E3 and NRL by week 17 of differentiation (Fig. 2D). Similarly, CRX, RCVRN and RHO, L/M OPSIN were detected in cells showing distinct photoreceptor cell morphologies in the presumptive ONL by week 33 of differentiation (Fig. 2E). By 36 weeks rod and cone cell segment-like structures contained phototransduction proteins including GNAT1, PRPH2 and protein ARL13B involved in development, function and maintenance of cilia^28,29^ (Fig. 2F).

### Establishment of xeno and xeno-free microdissection-free 3D retinal differentiation platform

**Figure 3:**
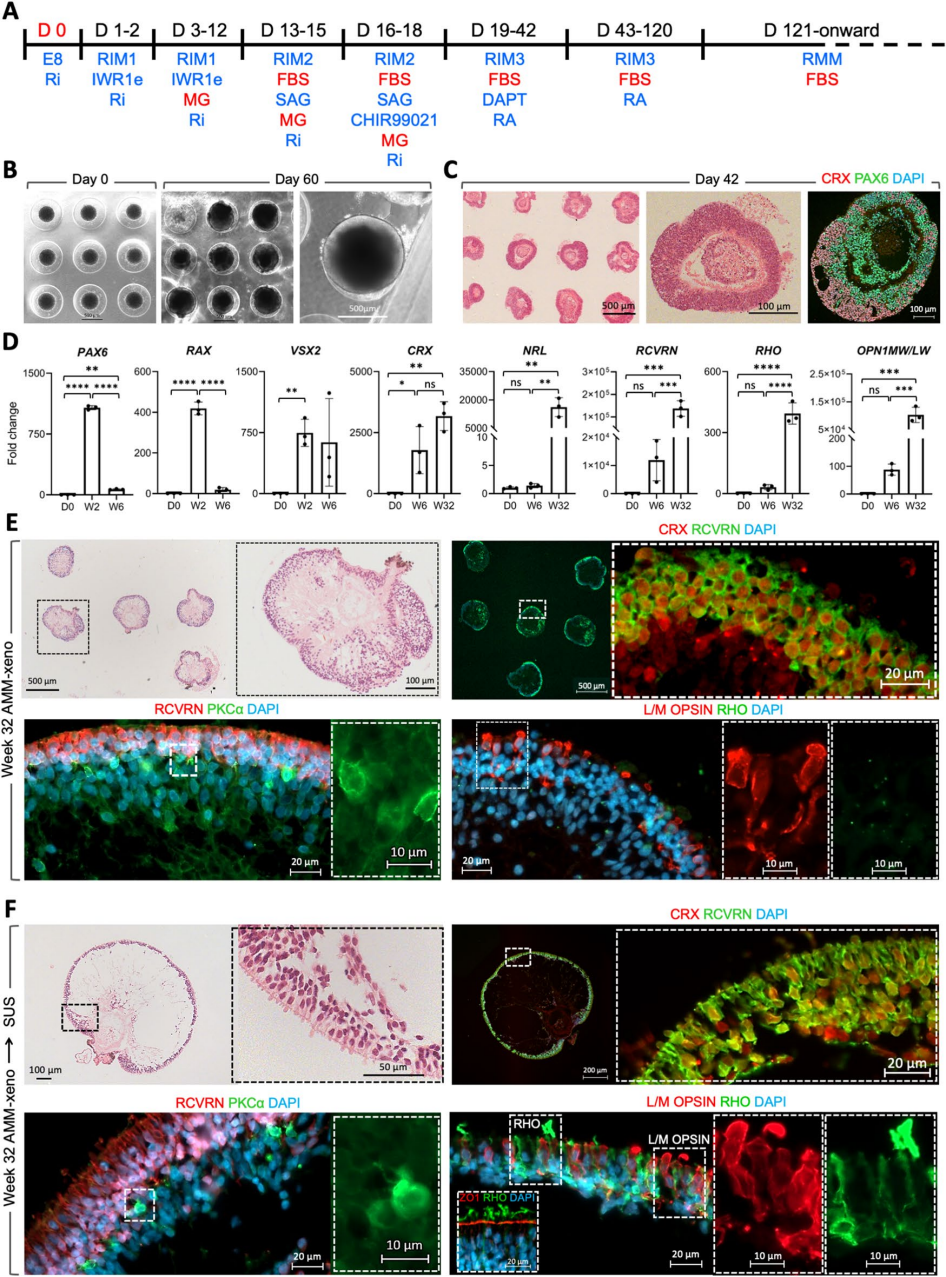
Retinal differentiation in 3D AMM using animal-derived supplements (xeno). (**A**) Schematic of the 3D AMM xeno small molecule protocol (**B**) Bright-field microscopy of aggregates generated from MShef10 ESC line in AMM at day 0 (first image from left) and differentiated 3D optic vesicle-like structures at day 60 of differentiation (scale bars 500 µm) (**C**) Histological and immunohistological analysis of CRX and PAX6 in retinal organoids generated from MShef10 ESC line at day 42 of differentiation. (**D**) RT PCR analysis of relative expression of retinal-specific markers (mean ± SD; n = 3 independently cultured AMMs each containing over 50 OVs from N = 1 ESC line, MShef10. (**E**,**F**) Histological and immunohistological analysis of CRX, RCVRN, L/M OPSIN, RHO and PKCa at week 32 of differentiation in retinal organoids generated from MShef10 ESC line. 3D retinal organoids which were transferred from AMM into suspension culture from week 6 of differentiation grew larger and maintained laminated organisation and exhibited longer outer segment-like protrusion beyond outer limiting membrane (**F**) compared to organoids which were maintained in AMM throughout the study (**E**).

Multi-step approaches to form and then manually micro-dissect the OVs are labour intensive and time consuming. Therefore, to reduce the labour-intensity of our 2D/3D small molecule directed differentiation protocol and to increase the capacity for OV production, we applied an agarose micromould (AMM) platform that standardised the generation of self-assembled 3D structures from dissociated hESCs (Fig. 3A). Each AMM contained 81 microwells of 800 µm in diameter and depth, with ease of scale up achieved by culturing AMMs in 12 well plates (972 microwells per plate), while individuals OVs within each AMM were interconnected via the culture media (Fig. 3B). Reproducible generation of OVs was observed in microwells and by H & E staining and IHC analysis of sectioned AMMs for CRX and PAX6 at week 6 of differentiation (Fig. 3C). RCVRN IHC at week 32 of differentiation showed 85.9 ± 2.5% of OVs were RCVRN + ve, with one OV usually forming per microwell (Mean ± SD of N = 3 differentiations analysing 124 organoids from ESC line MShef10). Supplementary Fig. 2 shows IHC analysis of a set of OVs from a single AMM showing RCVRN + ve cells. When viewed in cross-section, inter OV variation is seen, in some the retinal neuroepithelium extends around the vesicle (e.g. OV number 3, 14, 30), but commonly a RCVRN-ve stretch is also observed (e.g. OV number 3, 9). RT-PCR analysis confirmed early upregulation of *PAX6*, *RAX* and *VSX2* followed by expression of photoreceptor-specific genes *CRX*, *NRL*, *RCVRN*, *RHO* and *OPN1MW/LW* after extended differentiation (Fig. 3D) in line with retinal development. We compared culture of OVs from week 6 of differentiation either continued in AMMs (Fig. 3E), or after transfer to suspension cultures in poly 2-hydroxyethyl methylacrylate (pHEMA)-coated plates (Fig. 3F). By week 32 of differentiation OVs grown inside AMMs (Fig. 3E; 6/6 wells ~ 100%), were typically smaller, possibly due to the physical restraint of the 800 μm diameter microwells, compared to OVs matured in suspension (Fig. 3F). IHC analysis revealed that under both conditions a laminar retinal structure was generated with cells exhibiting spatiotemporal expression of retinal markers. CRX + ve and RCVRN + ve photoreceptors were predominantly located in the presumptive outer nuclear layer and PKCα + ve bipolar cells in the presumptive inner nuclear layer. OVs matured in suspension exhibited photoreceptors with an elongated morphology and frequent OS-like structures. Of note, RHO + ve rods were not detected in OVs matured inside AMMs, while formation of L/MO + ve cones was achieved under both conditions (Fig. 3E,F). To assess the retinal differentiation efficiency of the xeno-AMM system using different pluripotent stem cell lines, we determined the percentage of RCVRN + ve cells (Mean ± SD, 31.1% ± 17.4) by immunostaining of dissociated OVs from 5 independent xeno-AMM differentiation cultures from three different ESC cell lines (mShef10, MShef4, MShef12) at weeks 23–29 (Supplementary Fig. 3). Supplementary Fig. 4 shows additional examples of retinal organoids generated from two ESC lines showing phalloidin labelling of the outer limiting membrane at the organoid periphery and RCVRN + ve and ARR3 + ve cone photoreceptor cells located in the presumptive outer nuclear layer.

**Figure 4:**
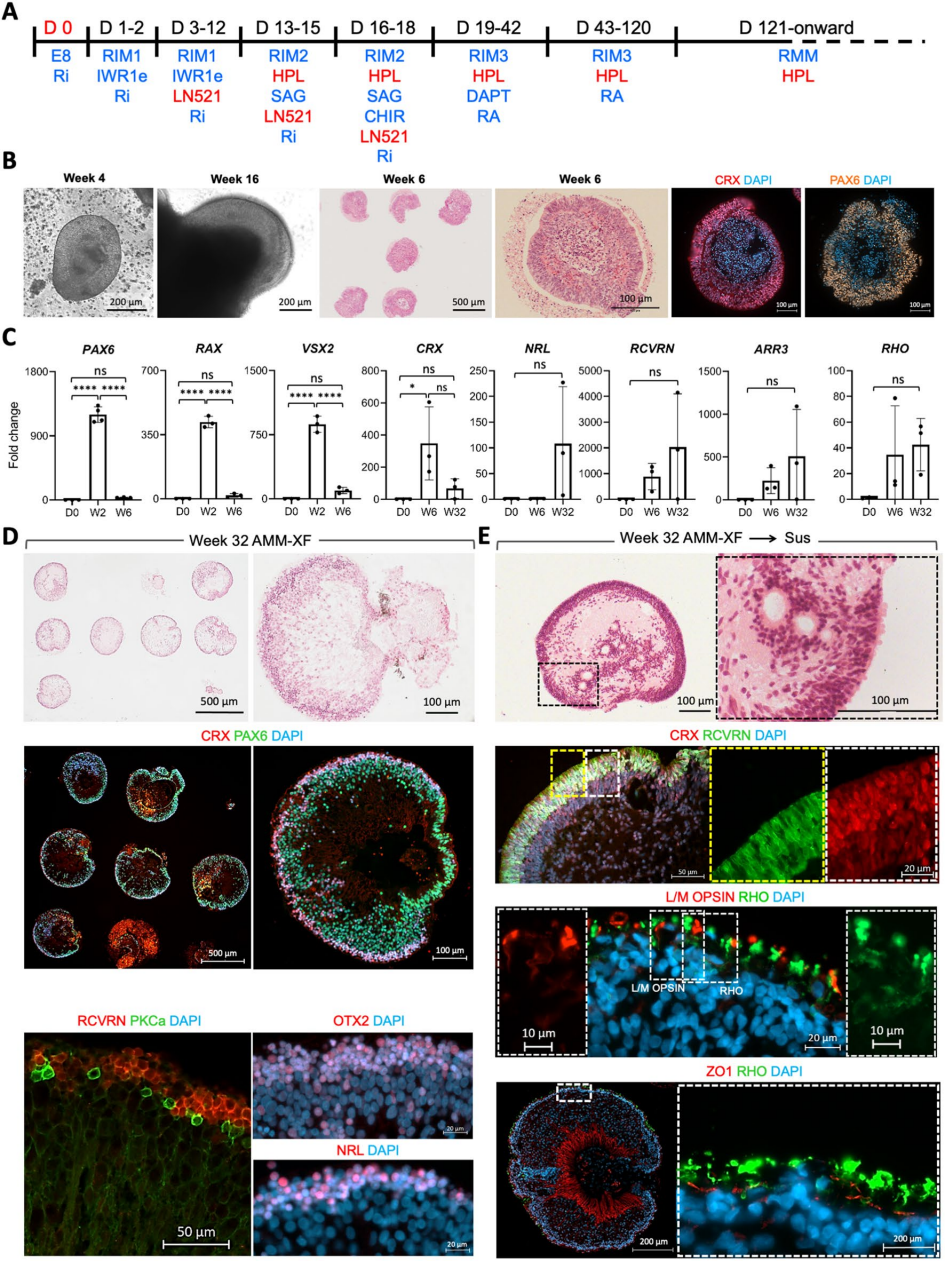
Retinal differentiation in 3D AMM without animal-derived supplements (xeno-free). (**A**) Schematic of the 3D AMM xeno-free small molecule protocol (**B**) Bright field images and immunohistological analysis of retinal organoids at wk 4, 6, and 16 of differentiation (**C**) RT PCR analysis of relative expression of retinal marker genes (mean ± SD; n = 3 independently cultured AMMs, each containing over 50 OVs from MShef10 ESC differentiation). (**D**,**E**) Immunohistological analysis of retinal organoids at week 32 of differentiation, maintained in AMMs (**D**) or transferred from AMM into suspension culture from week 6 of differentiation (**E**; Sus). Data shown from differentiations of MShef10 ESC line.

Finally, we examined the effect of xeno-free supplements on OV generation in AMMs, by substituting MG and FBS with rLN521 and HPL, respectively in the small molecule directed AMM differentiation protocol (Fig. 4A,B; referred to as AMM xeno-free). IHC localisation of CRX and PAX6 in sectioned OVs at week 6 of differentiation showed that the xeno-free supplementation supported retinal induction and specification (Fig. 4B). RT-PCR analysis confirmed expression of *PAX6*, *RAX*, *VSX2* and *CRX* followed by expression of *NRL*, *RCVRN*, *ARR3* and *RHO* after extended differentiation (Fig. 4C) albeit at relatively lower levels of fold change and with more variability between AMMs (p > 0.05) than observed in the AMM-xeno cultures. Comparison of levels of expression of photoreceptor-specific genes in independent sets of AMM organoids at week 28 of culture confirmed the higher expression levels in AMM xeno- compared with AMM xeno-free organoids, with cone markers particularly elevated, reminiscent of an early stage of retinal development (Supplementary Fig. 5). RCVRN IHC at week 32 of differentiation indicated a lower percentage of OVs were RCVRN + ve (71%, Supplementary Fig. 6) than under xeno-free conditions. These data, like the 2D/3D differentiations, showed both xeno and xeno-free conditions promoted upregulation of photoreceptor-specific genes but with more efficient differentiation in the xeno conditions.

We also investigated whether levels of RPE gene expression (*MITF*, *RPE65*, *BEST*) were relatively enhanced in the xeno-free AMM organoids (Supplementary Fig. 7), as in the 2D/3D xeno-free directed differentiation protocol expansion of presumptive RPE was observed following microdissection of OVs and transfer to U-bottom low-adherent 96-well plates (data not shown). Indeed RT-PCR analysis showed a marked upregulation of *MITF* expression in AMM organoids grown under xeno-free conditions at 6 weeks (Supplementary Fig. 7A). By week 32 both xeno and xeno-free AMM systems showed expression of mature RPE marker Bestrophin (BEST), with more sustained RPE65 expression in the xeno-free system (Supplementary Fig. 7A). AMM OVs typically form a 3D thin pigmented epithelial structure adjoining the neuroepithelium (Supplementary 7B, Fig. 3E,F; Fig. 4D). Under xeno-free AMM conditions in some organoids the pigmented epithelium appeared expanded (Supplementary 7B; not quantified). By week 32 of differentiation, RPE markers such as BEST, and ZO1 (tight junction protein-1) were detected by IHC (Supplementary Fig. 7B,C). In ultrathin sections at week 40 imaged by TEM, formation of microvilli (m), and melanosomes at different stages of maturation were observed indicating RPE differentiation occurring alongside photoreceptor cell differentiation (Supplementary Fig. 7D).

Organoids generated in AMM xeno-free cultures at later stages showed onward neural retinal tissue lamination with CRX, RCVRN NRL + ve photoreceptors localised in the outer nuclear layer and PKCα-labelling of bipolar cells (Fig. 4D). Transfer of OVs from AMMs to suspension culture under a xeno-free regimen, led to the development of OS-like structures containing rhodopsin (RHO) or L/M cone opsin protein by week 32 of differentiation (Fig. 4E).

**Figure 5:**
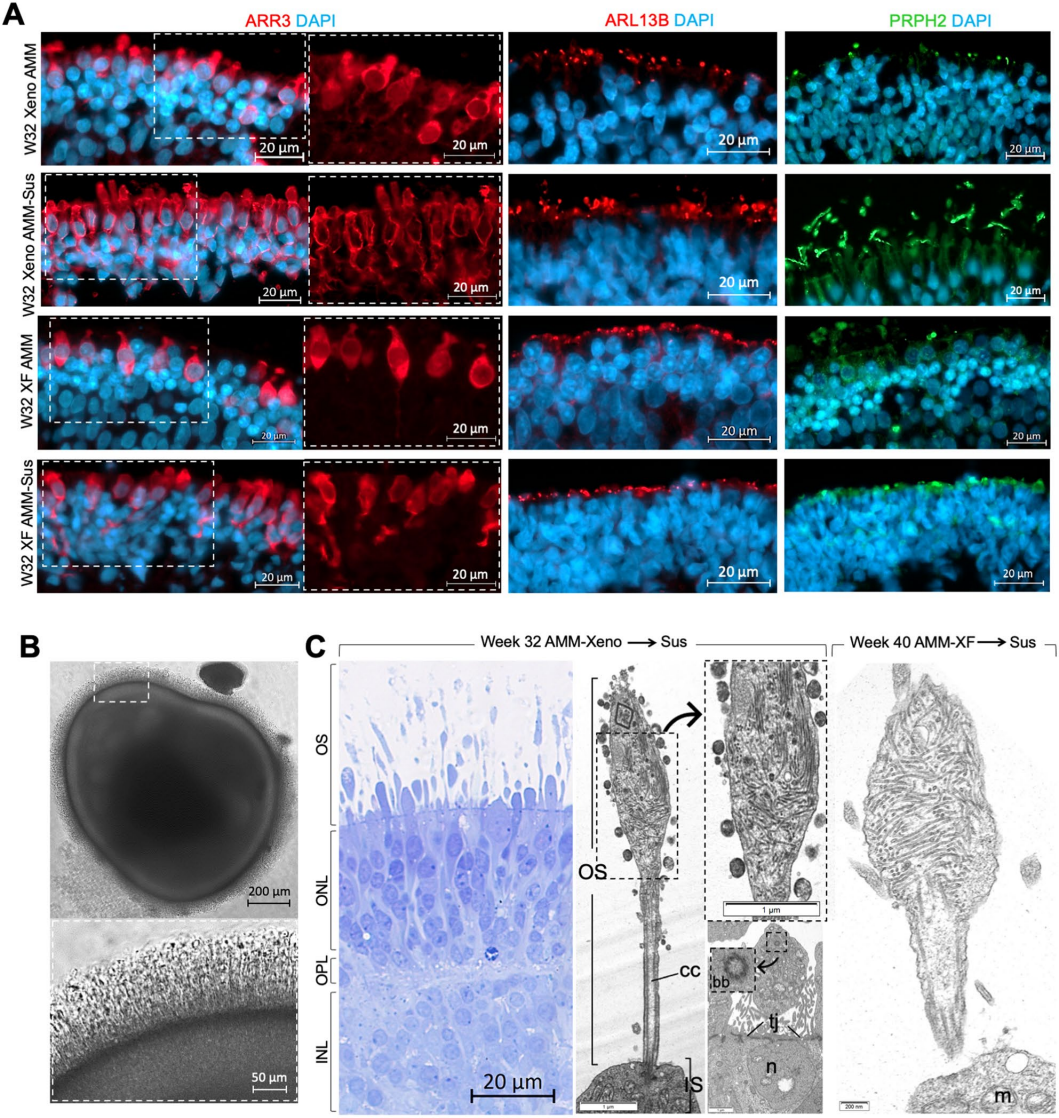
Formation of laminated retinal organoids with OS-like structures and expression of proteins associated with mature PRs. (**A**) Immunohistochemical staining of retinal organoids for ARR3, ARL13B, PRPH2 show morphological maturation and apical organisation of developing photoreceptor segment-like structures. Retinal organoids generated in AMMs using animal-derived supplements (xeno) or xeno-free (XF) conditions, transferred to suspension culture from day 60 of differentiation or maintained in AMMs showed immunostaining for cone outer segment protein ARR3. Retinal organoids matured in suspension in xeno conditions developed prominent OS-like structures labelling with ARR3, ARL13B or PRPH2. (**B**) Bright-field image of whole mount retinal organoid at week 32 of differentiation generated from MShef10 ESC line using AMMs and animal-derived supplements (xeno) and cultured in suspension; OS-like structures (arrowhead) developed from week 22–25 of differentiation. (**C**) IS, nascent OS, OLM, bb, cc and mt were detected in TEM images taken from ultrathin sections of week 32 retinal organoids (cultured in AMMs and grown in suspension under xeno conditions; n = 2) and of week 40 retinal organoids (AMMs in suspension under xeno-free conditions; n = 2) (MShef4 ESC line); cc, connecting cilia; OS, outer segment; IS, inner segment; bb, basal body; mt, mitochondria, OLM, outer limiting membrane, tj, tight junction.

IHC using a panel of markers to compare the morphology and organisation of photoreceptors generated in xeno or xeno-free conditions in retinal organoids cultured inside AMM and in suspension for an extended period of differentiation showed ARR3, PRPH2 and cilia marker ARL13B localised at the apical surface under all conditions (Fig. 5A). Photoreceptors in retinal organoids matured in suspension under xeno conditions typically exhibited more mature photoreceptor cell morphologies indicated by formation of OS-like protrusions beyond the outer limiting membrane (OLM; Fig. 5A) compared with the xeno-free suspension cultures. Continued culture inside AMM for 32 weeks appeared to limit morphological maturation and organoids showed shorter cell bodies and more rounded nuclei (Fig. 5A). TEM microscopy showed formation of inner (IS) and outer segments (OS), outer limiting membrane (OLM), cilia basal body (bb) and connective cilia (cc) in ultrathin sections (Fig. 5B,C and Supplementary Fig. 8) in organoids generated under xeno conditions (32 weeks), and under xeno-free conditions after extended cultures (40 weeks). These data indicate that the AMM platforms generate photoreceptor cells that exhibit correct orientation and morphological differentiation.

## Discussion

In this study, we describe four platforms, which generate 3D retinal organoids from human pluripotent stem cells using both animal-derived and animal-free supplements. Our study tested the platforms on human embryonic stem cells. In future studies it will be valuable to perform comparative studies of induced pluripotent stem cell lines, which can show more variability compared to ESC lines. Initially we developed an amended 2D/3D protocol (based on the Gonzalez protocol) by introducing a regimen of small molecules to induce directed differentiation of hPSCs into retinal tissue using animal-derived supplements. Directed differentiation promoted the faithful temporal sequence of retinal tissue formation mimicking human development and the resulting OVs contained retinal cells organised in multi-layered 3D structures. Generated photoreceptors exhibited key structural features of photoreceptor cells including the formation of OS-like structures containing proteins involved in phototransduction. Similar to previously published work^20,30^, this system supports generation of both cone and rod photoreceptors. As reliance on animal-derived supplements is a major bottleneck for clinical translation, we adapted the 2D/3D platform by direct substitution of MG and FBS with rhLN521 and HPL respectively and showed that OVs formed and matured successfully under xeno-free condition. HPL (human platelet lysate) is produced mostly from expired human platelet concentrate (hPC), derived from whole blood donation meant for blood transfusion. Good Manufacturing Practice (GMP)-compliant manufacturing protocols allow the production of clinical-grade HPL that can be used for expansion of human cells for cell therapy^31^. HPL application for wound healing and guided bone regeneration in periodontal regeneration surgery has been widely described and shown superiority over FBS^32^. Autologous HPL could also be preferential for personalised cell therapies^33^.

As microdissection has been typically required to isolate OV-like structures at earlier stages of differentiation in protocols developed so far, we developed a 3D platform using AMMs to circumvent the manual microdissection requirement and allow generation of OVs at scale. A similar hydrogel platform (using custom-made 1.5 mm diameter polyethylene glycol hydrogel milli-wells; 7 per culture vessel) was recently developed to generate retinal organoids from mouse ESCs^34^. In addition, AMMs have been used in various non retinal 3D platforms, and were used in a recent study that generated uniform sized EBs in a 3D/2D/3D platform to generate retinal organoids^26^. Cowan et al. plated out the EBs formed in AMMs and then manually detached adherent organoids using a checkerboard scraping technique. A similar approach of using a scraping method to release organoids from 2D cultures was also used by Regent et al., 2020^35^. After scraping cell clumps the latter used a pair of 30 gauge / 0.5 inch hypodermic needles fitted to 1 ml syringes to dissect bunched optic vesicles using a scissoring motion under a stereomicroscope. Both of these protocols also use Matrigel. Here, we developed a novel 3D platform using AMMs to generate human retinal organoids, which does not require manual micro-dissection and allows large-scale generation of retinal organoids. Our platform does not involve a 2D culture step, instead suspended organoids form within agarose micromold wells. Standardised generation of OVs and differentiation of photoreceptors is also highly desirable for retinal disease modelling^36^. Generation and maturation of retinal organoids was achieved efficiently using animal-derived supplements, and was also demonstrated under xeno- and serum-free conditions. In this study we focussed on photoreceptor cell production within the organoids rather than inner retinal cells. While the photoreceptor layer is typically preserved after extended culture, we noted that INL and RGC lamination is variable (Supplementary Fig. 9 shows examples of OVs cultured in xeno-free conditions showing variability of IHC with beta-tubulin, TUBB3, which labels retinal ganglion cells). Lack of RGCs in late-stage retinal organoids has been previously observed, with a recent study showing decline in RGC number from 18 to 24wks of culture^26^. Although the timing of OV appearance remained similar under xeno-free conditions, expansion of presumptive RPE was more apparent compared to cultures supplemented with animal-derived supplements; it is possible that further optimisation may reduce differentiation shifting towards RPE at the expense of photoreceptor differentiation. The limited physical space in each well of AMMs appeared to hinder OVs full maturation, as evident by lack of RHO protein localisation by IHC, and improved maturation was achieved by transfer to suspension for long term culture. In conclusion, the AMM platform provides a useful scalable system to generate retinal organoids and rod and cone photoreceptors expressing pan-photoreceptor markers and rod- and cone-specific markers.

## Experimental procedures

### Maintenance of human PSCs

hPSC lines, including three embryonic stem cell lines (MShef4, MShef10 & MShef12) generated by the Centre for Stem Cell Biology, University of Sheffield^37^ were cultured on recombinant laminin-521 (rhLN-521, BioLamina)-coated plates in serum-free Essential 8 (E8) medium (ThermoFisher Scientific). To coat the plates, 5 µg/ml solution of rh-LN521 in PBS containing Ca^2+^ and Mg^2+^ was used. Cells were fed daily and passaged when 70–80% confluent using 0.5% EDTA solution (Sigma Aldrich) and replated at 1:4 ratio. The cell lines were monitored regularly for mycoplasma infection and were propagated in antibiotic free medium. Representative images in the paper were from MShef10ESC line unless stated otherwise.

### 2D/3D small-molecule-based retinal induction

Stepwise differentiation was initiated when hPSC cultures become 80% confluent in 6-well plates. Retinal Induction Medium 1 (RIM1) composition can be found in supplementary materials. Briefly it contained G-MEM BHK-21, Knockout Serum Replacement, GlutaMAX, Na-Pyruvate, 2 mercaptoethanol and Penicillin and Streptomycin (P/S). Following washing with RIM1, cells were cultured with RIM1 supplemented with 3 µM IWR1e (Merck Millipore) and 6 µl/ml Matrigel (Corning). The Supplemented RIM1 medium was changed every 48 h for 8 days. On day 8 of differentiation the cells were washed gently with RIM2-xeno and cultured for 3 more days with RIM2-xeno (RIM1 plus 10% foetal bovine serum, FBS, Thermo Fisher Scientific) supplemented with 100 nM Smoothened agonist (SAG, Merck Millipore) and 6 µl/ml Matrigel followed on day 12 by 3 days culture with RIM2-xeno supplemented with 100 nM SAG, 3 µM CHIR99021 (Merck Millipore) and 6 µl/ml Matrigel. On day 15, cells were washed with RIM3-xeno and cultured for 10 days (medium change every 48 h) with RIM3-xeno supplemented with 0.5 µM RA (Sigma Aldrich) and 10 µM DAPT (Sigma Aldrich). RIM3-xeno contained DMEM-F12, N2, FBS, MEM-NEAA, Taurine and P/S (composition can be found in supplementary materials). From day 25 of differentiation, cells were cultured for 36 more days with RIM3-xeno supplemented with only 0.5 µM RA. On day 61 of differentiation, retinal organoids were washed and cultured with Retinal Maturation Medium-xeno (RMM-xeno) without RA to further differentiate retinal organoids. RMM-xeno contained G-MEM BHK-21, B27, FBS, Na-Pyruvate, Taurine and P/S (composition can be found in supplementary materials). From day 18 onward, self-organised 3D structures started to emerge from the adherent culture. At day 30, prominent 3D organoid structures were microdissected using a G30 needle under a microscope in the hood and transferred individually (manually lifted) into a low adherent U-bottom 96-well plates. After dissection organoids were maintained in 96-well plates, one organoid per well. Animal-free supplementation was used to generate retinal organoids under xeno- and serum-free condition using exactly the same small molecule regimen but substituting 10% FBS for 1% Human Platelet Lysate (HPL, StemCell Technologies) and 6 µl/ml Matrigel for 6 µl/ml rhLN-521. The composition of various culture media can be found in supplementary data.

### Formation of self-aggregated 3D-hPSCs spheroids in AMM

The AMMs were manufactured in 81-well format using the 3D PetriDish® mould (Sigma Aldrich) using 2% low-melting temperature agarose suitable for cell culture (Sigma Aldrich) following the manufacturer’s instructions and transferred into 12-well plates (Corning). Scaled up hPSCs on rhLN521-coated culture dishes were incubated with 1 ml of Gentle Dissociation Buffer (STEMCELL Technologies) for 7–10 min at 37 ˚C. Following this, single cell suspensions were prepared by pipetting the buffer up and down gently. The cell suspension was centrifuged at 0.2 RCF for 5 min and resuspended in E8 supplemented with 10 µM Y-27632 (Cayman Chemical Company) at a density of 3.84 × 10^6^ live cells/ml. The AMMs were seeded by transferring 190 µl of cell suspension onto each 81-well AMM (~ 729,000 cells were seeded per AMM according to the manufacturer’s instructions, 3D PetriDish® mould (Sigma Aldrich) leading to ~ 9,000 cells sedimenting into each well to form a spheroid). After 2–3 h, 1 ml E8 supplemented with 10 µM Y-27632 were gently added to each well of 12-well plate and incubated overnight.

### Retinal induction of self-aggregated PSCs-derived 3D spheroids

Differentiation was initiated on day 1 by replacing E8 with RIM1 supplemented with 3 µM IWR1e and 20 µM/ml Rock inhibitor for two days. Then the culture medium was replaced on day 3 with RIM1 supplemented with 3 µM IWR1e, 20 µM/ml Rock inhibitor and 6 µl/ml Matrigel. The medium was changed every 48 h for 10 days. On day 13, the medium was replaced with RIM2-xeno supplemented with 100 nM SAG and 6 µl/ml Matrigel and 20 µM/ml Rock inhibitor for 2 days followed on day 15 by 3 days culture with RIM2-xeno supplemented with 100 nM SAG, 3 µM CHIR99021 and 6 µl/ml Matrigel. On day 19, medium was replaced with RIM3-xeno supplemented with 0.5 µM RA and 10 µM DAPT for an additional 24 days. From day 43 of differentiation, cells were cultured with RIM3-xeno supplemented with only 0.5 µM RA. From day 121 of differentiation, retinal organoids were cultured with RMM-xeno without RA to further differentiate retinal organoids. Retinal organoids can be maintained in AMMs for the entire duration or transferred to separate wells from week 6 onwards. For derivation of xeno-free organoids in AMMs, exactly the same small molecule regimen was used except 6 µl/ml Matrigel and 10% FBS was replaced with 6 µl/ml rhLN-521 and 1% HPL, respectively.

### Histology and immunofluorescence

3D retinal organoids were fixed in ice-cold methanol for 30 min, then washed in PBS and embedded in 2% Low-melting temperature Agarose (Sigma Aldrich). Agarose-embedded organoids were dehydrated through graded ethanol (EtOH) and embedded in paraffin wax to obtain sections with 4 µm in thickness. Antigen retrieval was performed on dewaxed and rehydrated slides (in xylene and descending graded EtOH) in 1 × Tri-sodium citrate buffer solution (2.94 g in 1L dH_2_O) containing 0.5% Tween20 (pH 6.0) for 20 min in a microwave. Washed slides were used for subsequent staining.

Paraffin-embedded 4-μm retinal organoid sections were stained after antigen retrieval with Eosin and Haematoxylin and mounted in Pertex mounting media (Thermo Fisher Scientific) before microscopy. Brightfield images were taken using a Zeiss Axioplan microscope and processed using ImageJ software.

For immunostaining, sections were blocked with 10% BSA in PBS containing 0.5% Tween 20 (PBST) and incubated with primary antibody overnight at 4˚C and were detected using species-specific fluorescent-conjugated secondary antibody (Alexa Fluor 488/Alexa Fluor 568/ Alexa Fluor 647; Invitrogen). Sections were counterstained with DAPI (4′,6-diamidino-2-phenylindole) and mounted with ImmunoFluoroMount™ (GeneTex) before microscopy using a ZeissObserver 7. The obtained images were processed using ZEN 2.6 Blue Edition Software. A list of antibodies used in this study can be found in Supplementary Table 1. The criteria for quantification of *RCVRN* + organoids was the detection of RCVRN + ve cells by IHC in a 4-μm thick paraffin-embedded cross-section of each organoid.

Cryo-sectioning of OVs fixed in PBS/4% PFA and immunohistochemistry (IHC) was performed as previously described^27^. Sections of > 3 independently cultured OVs were analysed for each antibody. Immunocytochemistry of dissociated OVs was performed as previously described^38^ with the following modification. hPSC-derived OVs were dissociated into a cell suspension using a papain-based, enzymatic method according to the manufacturer's instructions (Worthington Biochemical, Lorne Laboratories, U.K.) and spun down at 300 g for 5 min and plated on laminin-coated chamber slides (Labtec) or plastic 96-well plates with RMM-xeno medium. After 24–48 at 37 °C adherent cells were washed once with PBS and fixed with 4% PFA/PBS for 10 min at room temperature. After three washes with PBS, samples were blocked in 3% BSA, 0.1% (vol/vol) Triton X-100 in PBS for 1 h at room temperature. Blocking solution was replaced with primary antibody (Rabbit anti-RCVRN 1: 1000, AB85585, Merck Millipore) in 3% BSA, 0.1% (vol/vol) Triton X-100 in PBS overnight. After three washes with PBS, adherent cells were incubated for 1 h at room temperature with secondary antibody diluted in blocking solution (1: 500, Invitrogen, Goat anti-rabbit IgG Alexa Fluor 488 or 594) and counter stained for 5 min with DAPI (Sigma-Aldrich). Samples were imaged on Zeiss Observer 7 colour fluorescent microscope. The percentage of positive cells for RCVRN was established by counting > 120 DAPI stained cells from 3 fields of view for each differentiation condition.

### qRT-PCR

RNA was extracted from 3D retinal organoids using a Direct-zol™ RNA MiniPrep Plus extraction kit (Zymo Research). RNA quantity and quality were assessed using a Nanodrop system (Themo Fisher Scientific). Following this, cDNA was amplified using the RT^2^ First Strand Kit (RevertAid First Strand cDNA Synthesis Kit, Thermo Fisher Scientific) following the manufacturer’s instructions. qPCR was performed with TaqMan Fast Advance Mastermix and primer pairs listed in Supplementary Table 1 and analysed using a StepOnePlus™ Real-Time PCR System (AppliedBio systems™). Gene expression was normalised to glyceraldehyde 3-phosphate dehydrogenase (*GAPDH*) and expressed as relative expression over 3D aggregates (spheroids) on day 0 of differentiation (self-aggregated spheroids) in AMM culture experiments, and over undifferentiated hPSCs (~ 80% confluent culture) in 2D/3D culture experiments, using a cut-off Ct value of 40. qPCR was performed in triplicate and data analysis using *GAPDH* and Ct values was performed as previously described^39^ using GraphPad Prism software (version 8.0). Differentiation experiments for each protocol were analysed as separate data sets.

### Transmission electron microscopy

Transmission electron microscopy (TEM) was used to image the outer segment-like structures in organoids from two AMM xeno and one AMM xeno-free differentiation. Retinal organoids were fixed by glutaraldehyde fixative suitable for electron microscopy. A JEOL JEM-1400 120 kV Transmission Electron Microscope (JEOL UK Ltd., Welwyn Garden City) with a Deben AMT XR80 digital camera (Deben UK Ltd., Bury St Edmunds, UK) was used to obtain images from ultrastructures within retinal organoids.

### Statistics

Data were analysed by GraphPad Prism (version 8.0). Statistical methods were not used to predetermine sample size, there was no randomization designed in the experiments, and the studies were not blinded. Significance was determined by One-way analysis of ANOVA with Tukey’s multiple comparisons test with a single variance. Data are represented as mean ± SEM, * = p < 0.05, ** = p < 0.01, *** = p < 0.001. **** = p < 0.0001.

## Supplementary Information

Supplementary Information.
