# Supplementary material for: Generation of 3D retinal tissue from human pluripotent stem cells using a directed small molecule-based serum-free microwell platform
Source: Sci Rep. 2022 Apr 22;12:6646. doi: 10.1038/s41598-022-10540-1 (PMC9033780; doi:10.1038/s41598-022-10540-1)
Supplement: Supplementary file 1 — Supplementary Information. [file 41598_2022_10540_MOESM1_ESM.pdf]

Supplementary File

Supplementary Figure 1:

A) Image of retinal organoid generated from MShef4 ESC line using 2D/3D method with animal-derived supplements (xeno) and cultured for 32 weeks in suspension. Multi-layered retinal structures with OS-like protrusions (OS), outer (ONL), inner (INL) and ganglion cell layers (GCL). Synaptophysin (SYN) is detected mainly in layers representing outer (OPL) and inner plexiform layers (IPL). ZO1 marks the outer limiting membrane (OLM). **B)** Brightfield microscopy of a thin section prepared from retinal organoid generated from MShef10 hESC line at week 32 of differentiation. Formation of outer limiting membrane (OLM) and inner (IS) and outer segments (OS) can be distinguished. N.B. INL and GCL integrity is variable within and between organoids after extended cultures.

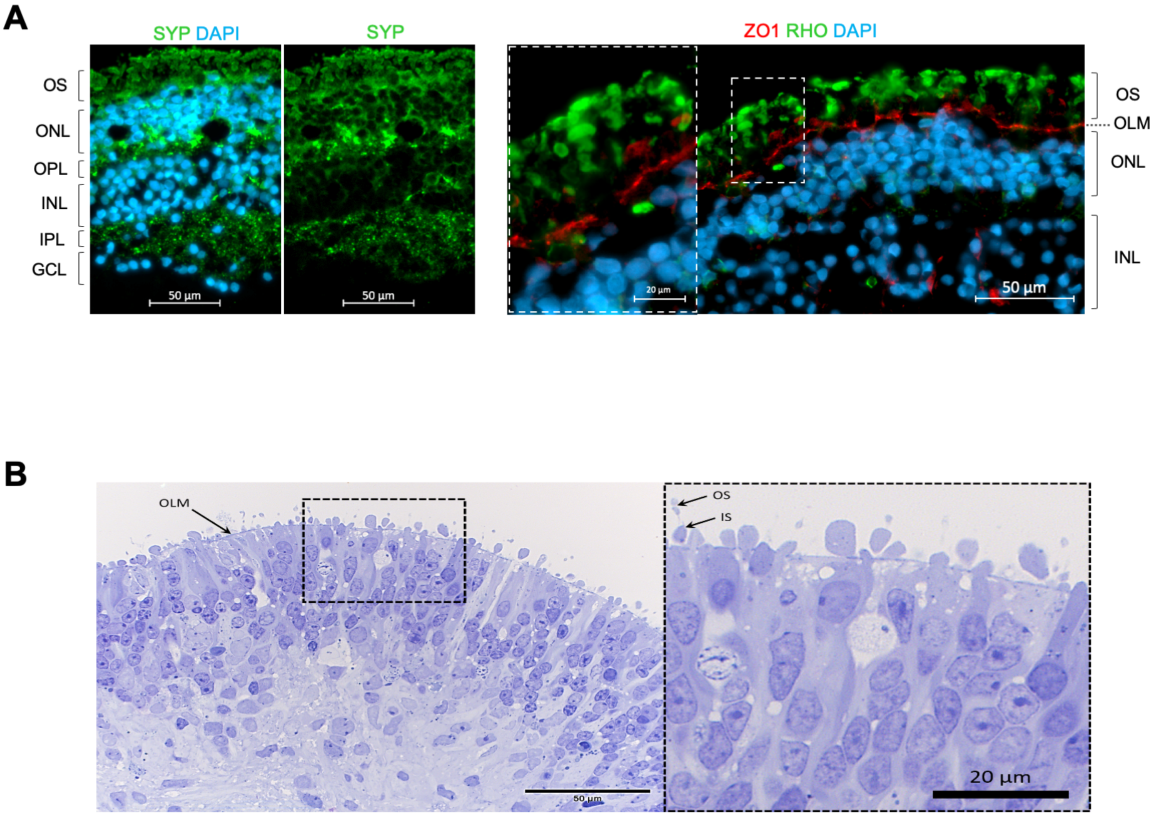

Figure S1

Supplementary Figure 2:

Retinal differentiation in 3D AMM xeno conditions. Immunohistochemical analysis of RCVRN (green) localisation in retinal organoids generated from MShef10 ESC. DAPI (blue). RCRVN+ organoids are numbered in white (1-32) and RCVRN -ve in orange (1-3). OVs typically show RCVRN+ve stretches of retinal neuroepithelium. Viewed in cross-section, inter OV variation is seen, in some sections the retinal neuroepithelium extends around the vesicle (e.g. OV number 3, 14, 30) in others a RCVRN-ve stretch is also observed (e.g. OV number 3, 9) and in others RCVRN+ve cells appear disordered (OV number 13).

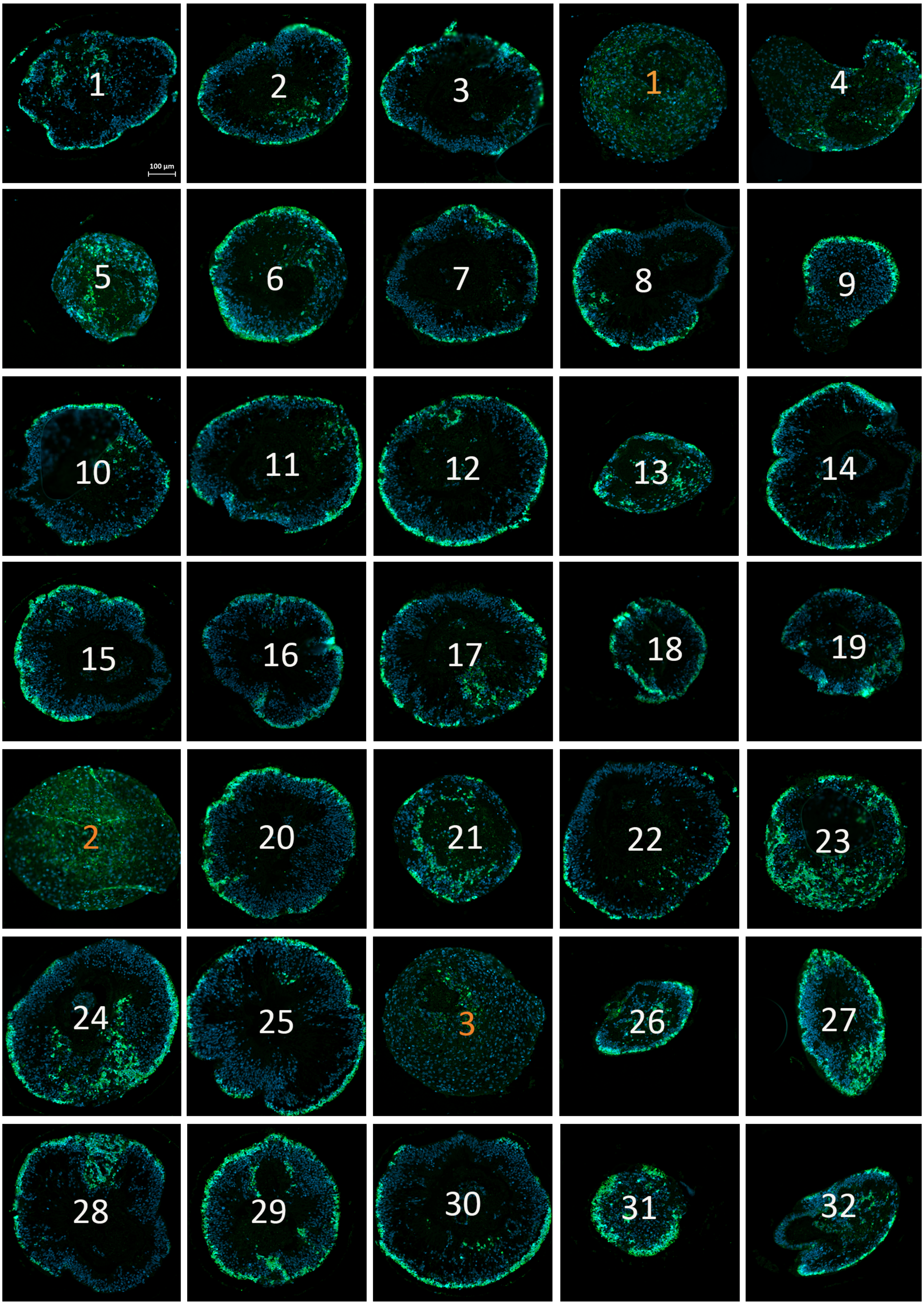
1
100 µm
2
3
1
4
5
6
7
8
9
10
11
12
13
14
15
16
17
18
19
2
20
21
22
23
24
25
3
26
27
28
29
30
31
32

Figure S2

Supplementary Figure 3:

Retinal differentiation in 3D AMM xeno conditions: efficiency of photoreceptor generation of the xeno-AMM system using different pluripotent stem cell lines.

A, B & C) Representative images of RCVRN immunostaining of dissociated cells from MShef10 (A), MShef12 (B) and MShef4 (C)-derived OVs. Arrow in B indicates inset boxed region shown at higher magnification. A', B' & C') Images of MShef10 (A'), MShef12 (B') and MShef4 (C')-derived retinal organoids cryo-sections stained by RCVRN (photoreceptor marker). D) Quantification of percentage RCVRN+ve cells in dissociated retinal organoid cells. N = 5 independent xeno-AMM differentiation cultures at weeks 23-29 from three different human ESC cell lines (MShef10, 20% RCVRN+ve, 32/160 cells; MShef10, 30%, 38/128 cells; MShef10, 46%, 120/260; MShef12 (blue), 9%, 17/187 cells and MShef4 (green), 51%, 135/267 cells). DAPI (blue) nuclear stain. Scale bar: 50 μm.

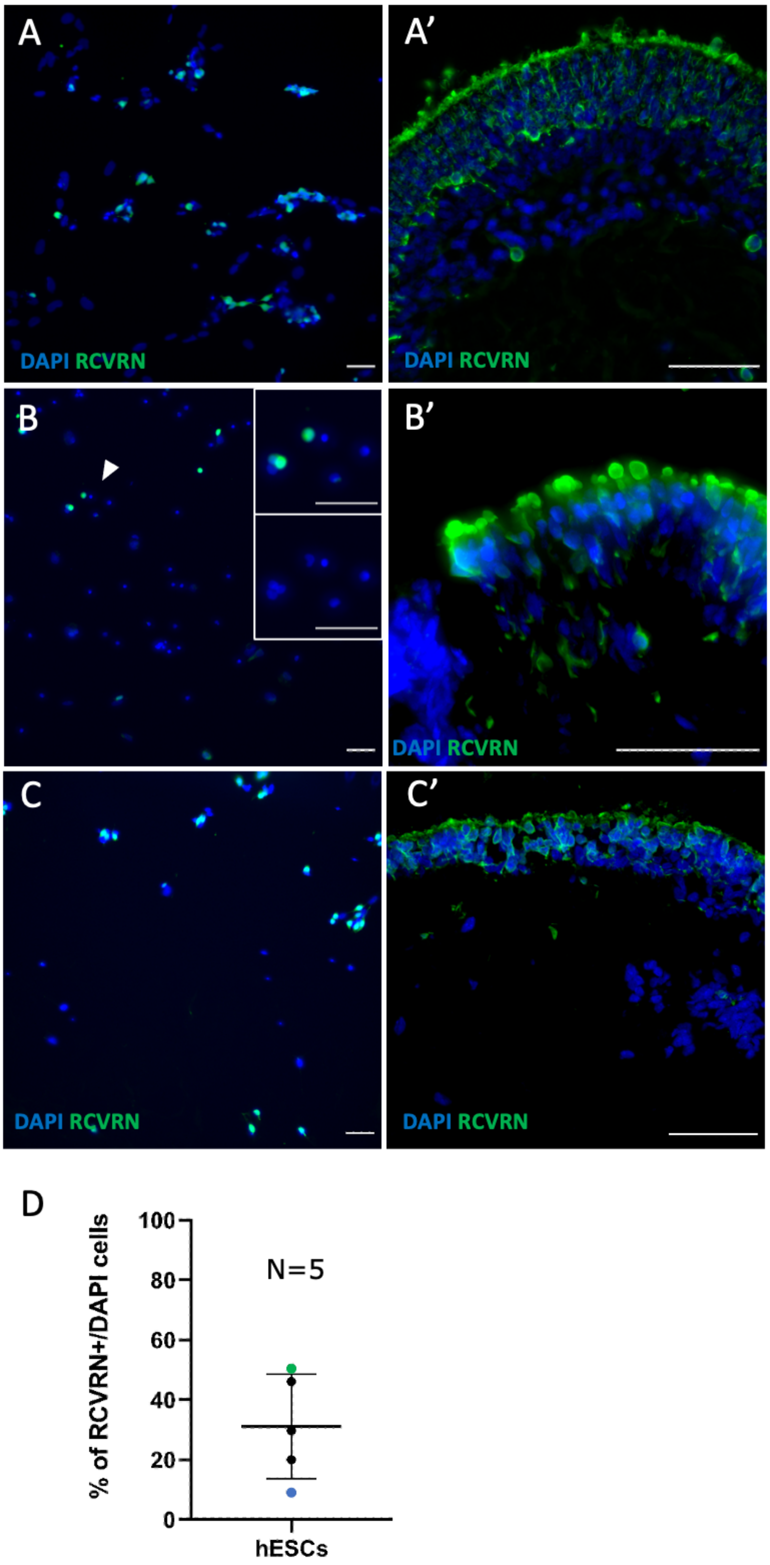
A
A’
DAPI RCVRN
DAPI RCVRN
B
B’
DAPI RCVRN
C
C’
DAPI RCVRN
DAPI RCVRN
D
% of RCVRN+/DAPI cells
100
80
60
40
20
0
N=5
hESCs

Figure S3

Supplementary Figure 4:

Retinal differentiation in 3D AMM xeno conditions: Retinal organoids generated from two independent ESC lines, MShef10 and MShef4.

A) IHC for RCVRN (yellow) labels photoreceptors localized at the periphery of organoids covering most of the OV perimeter. Scale bar: 200 μm. B) Immunostaining for phalloidin and ARR3 (cone arrestin) labels the outer limiting membrane and cone photoreceptor respectively, showing photoreceptor cell morphologies polarized with nascent segment like protrusions at the apical surface of the retinal organoid. Scale bar: 100μm.

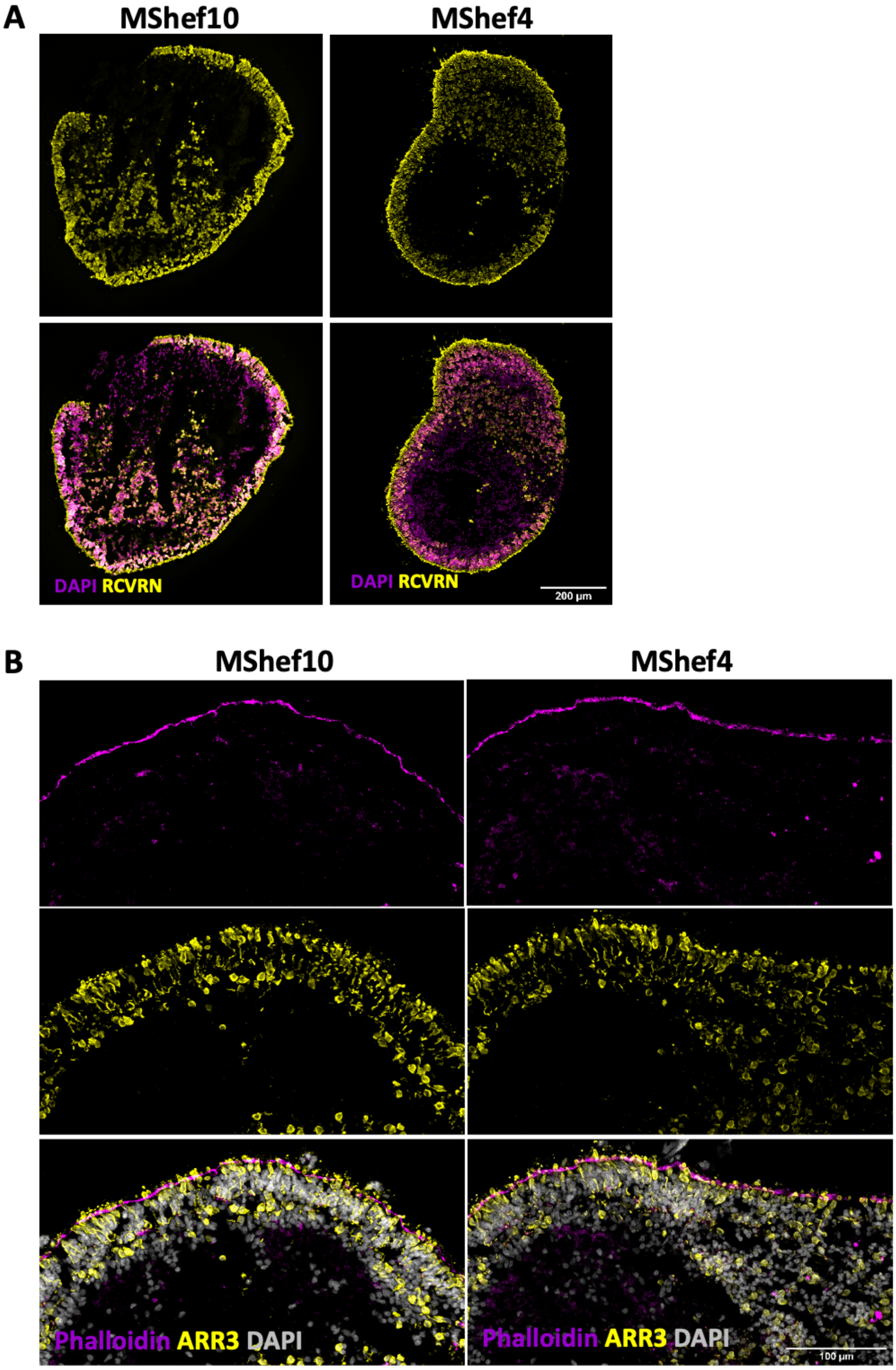
A
MShef10
MShef4
DAPI RCVRN
DAPI RCVRN
200 µm
B
MShef10
MShef4
Phalloidin ARR3 DAPI
Phalloidin ARR3 DAPI
100 µm

S

Figure S4

Supplementary Figure 5:

qRT PCR analysis of organoids cultured in 3D AMM xeno versus xeno-free conditions at week 28 showing relatively lower expression of photoreceptor (*RCVRN* & *CRX),* rod (NRL and RHO) and cone (*ARR3, OPN1SW & OPN1LW)* photoreceptor markers in xeno-free compared to xeno conditions. Fold change ± SD; n= 3 sets of 3 OVs from N=1 differentiation of MShef10 ESC line. Three independent passages of MShef10 ESC were used as undifferentiated control and two independent human adult retinal samples as positive control.

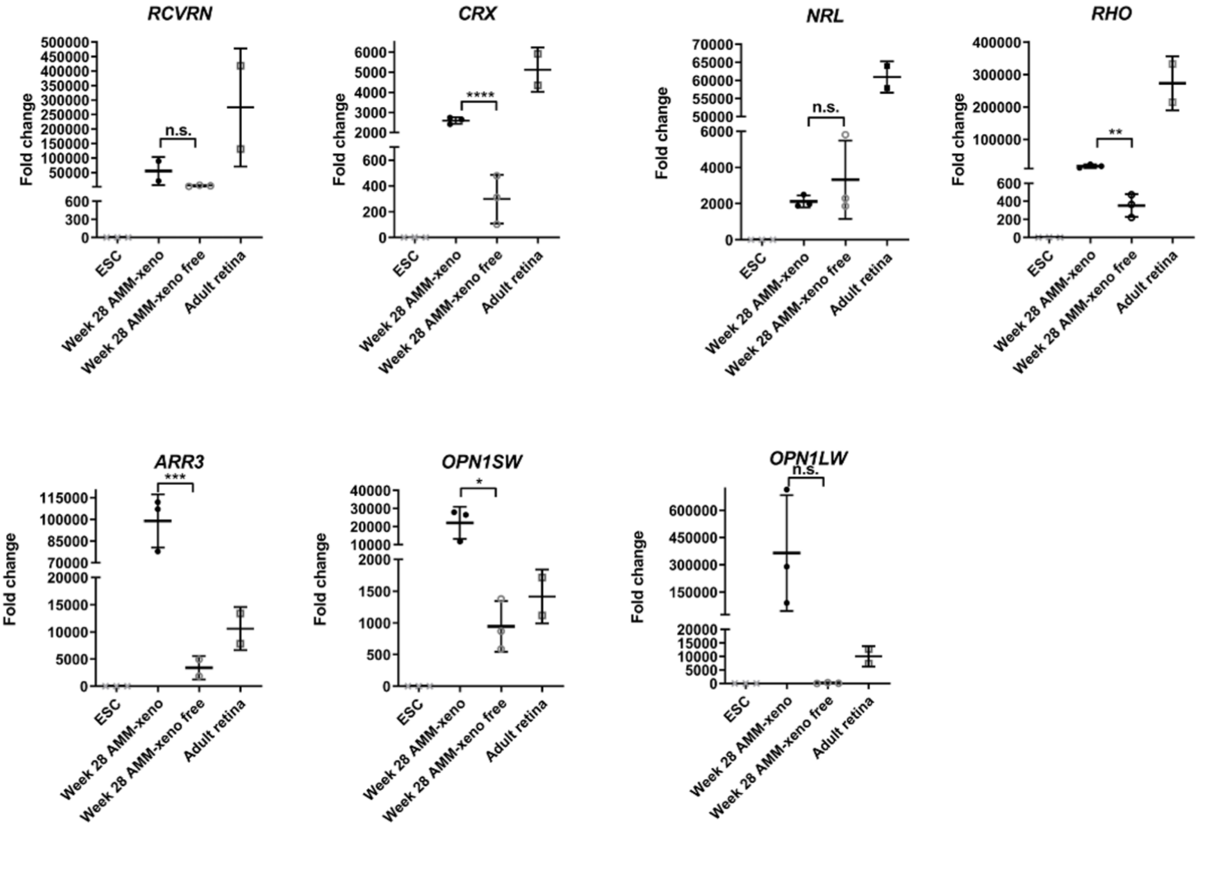

Figure S5

Supplementary Figure 6:

Retinal differentiation in 3D AMM xeno-free conditions. Immunohistochemical analysis of RCVRN (green) localisation in retinal organoids generated from MShef10 ESC line. DAPI (blue). RCRVN+ organoids are numbered in white (1-22) and RCVRN -ve in orange (1-9). 71.0% (22/31) of OVs showed RCVRN+ve cells typically organised in stretches of retinal neuroepithelium. Viewed in cross-section, inter OV variation is seen, in many the RCVRN+ve retinal neuroepithelium extends only part way around the vesicle (e.g. OV number 5, 18) and others show disordered RCVRN+ve cells (e.g. OV number 9). RCVRN-ve organoids are numbered in orange (OVs 1- 9).

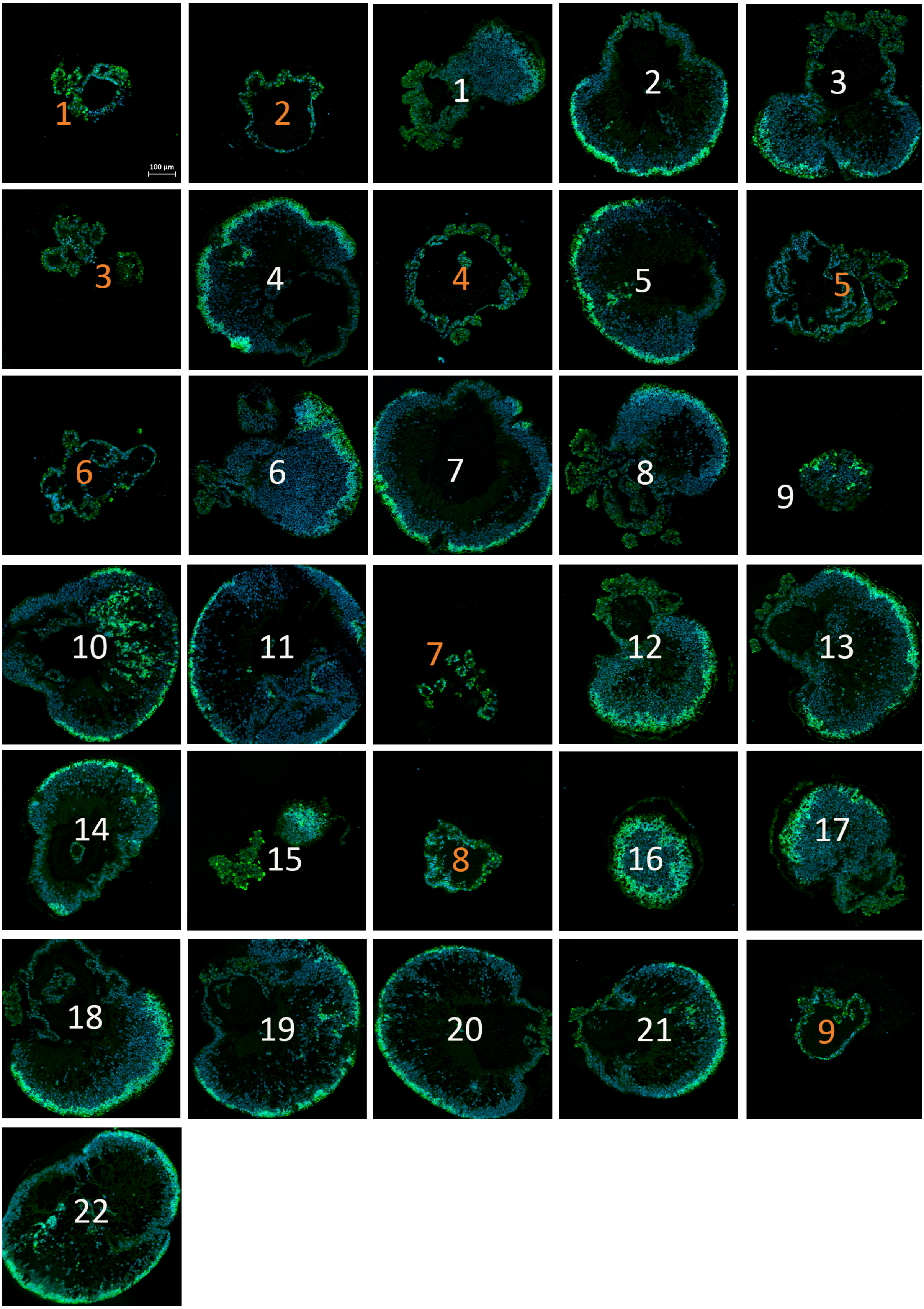

Figure S6

Supplementary Figure 7:

Pigmented epithelium in retinal organoids generated in 3D AMMs (xeno and xeno-free).
A) RT PCR analysis of relative expression of RPE markers (mean ± SD; n=3 AMMs each containing over 50 OVs from xeno and xeno-free AMM differentiation of MShef10 ESC line, maintained in AMMs until week 6 of differentiation and thereafter in suspension until week 32 of differentiation. **B)** H&E staining of week 17 & 32 organoids generated from MShef10 ESC line grown in suspension in xeno (top) or xeno-free (bottom) conditions after transferring from AMMs at week 6 of differentiation show pigmented (black/brown) epithelial cells organised as a 3D monolayer. In the top panel a thin pigmented layer is seen extending from a non-pigmented thickened neural retinal epithelium of the organoid **C)** Immunohistological analysis of retinal organoids at day 120 of differentiation generated in AMMs from MShef10 ESC line and grown in suspension under xeno-free condition showing retinal pigmented epithelium (RPE) marker BEST and ZO1 labelling of tightly connected RPE cells. **D)** TEM microscopy of pigmented monolayer showing formation of microvilli (mv) at apical surface, abundant melanosomes at different stages of maturation.

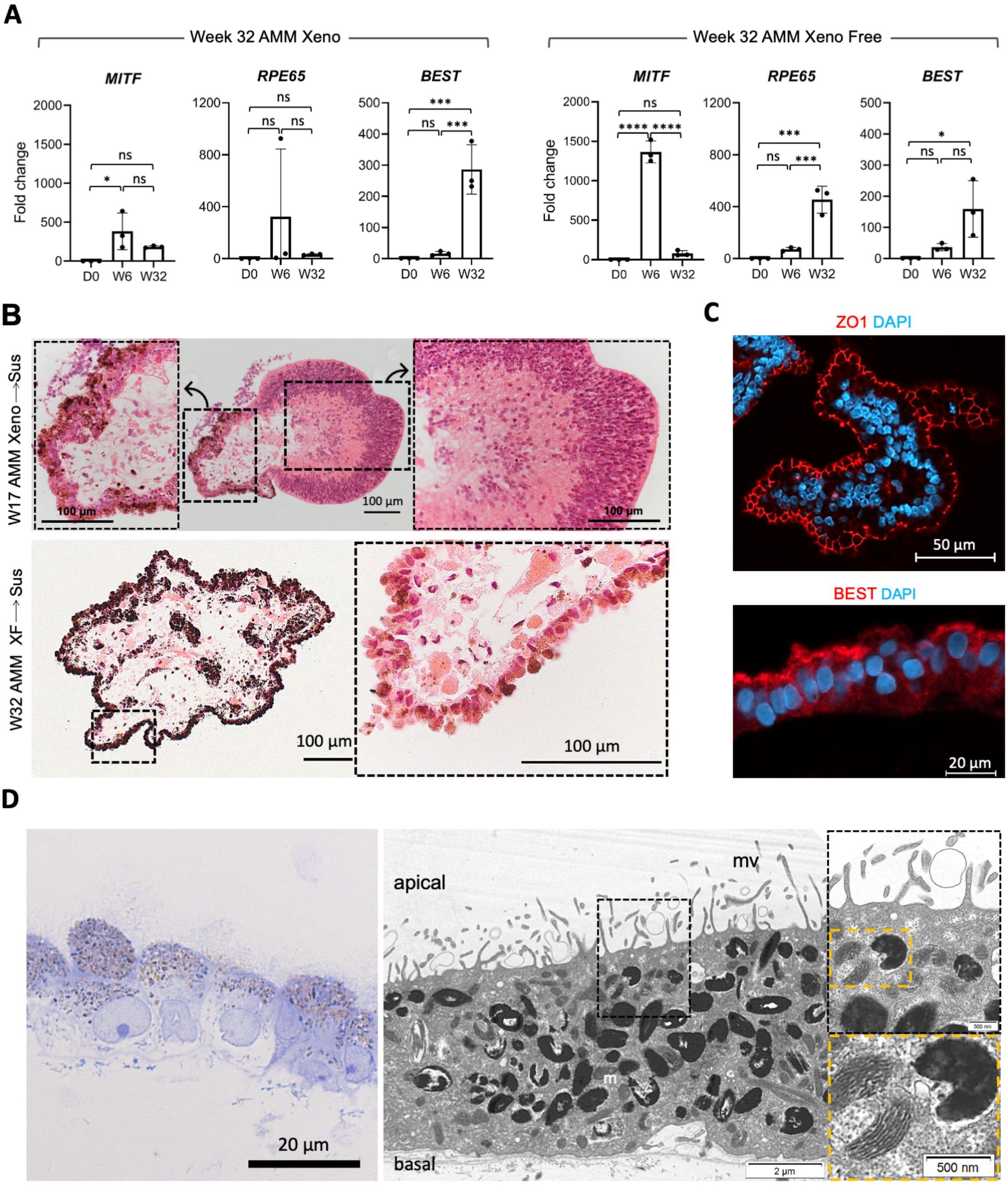
A
Week 32 AMM Xeno
Week 32 AMM Xeno Free
MITF
RPE65
BEST
Fold change
D0
W6
W32
ns
B
W17 AMM Xeno → Sus
W32 AMM XF → Sus
100 µm
C
ZO1 DAPI
50 µm
BEST DAPI
20 µm
D
20 µm
apical
basal
mv
m
2 µm
500 nm

Figure S7

Supplementary Figure 8:

TEM images taken from ultrathin sections obtained from week 32 retinal organoids (cultured in AMMs and grown in suspension under xeno conditions (MShef4 ESC line). Formation of IS, nascent OS, OLM, bb, cc and mt was detected. [Abbreviations: connecting cilia, cc; Outer segment, OS; Inner segment, IS; Basal body, bb; Mitochondria, mt, Outer limiting membrane, OLM, Tight junction; tj].

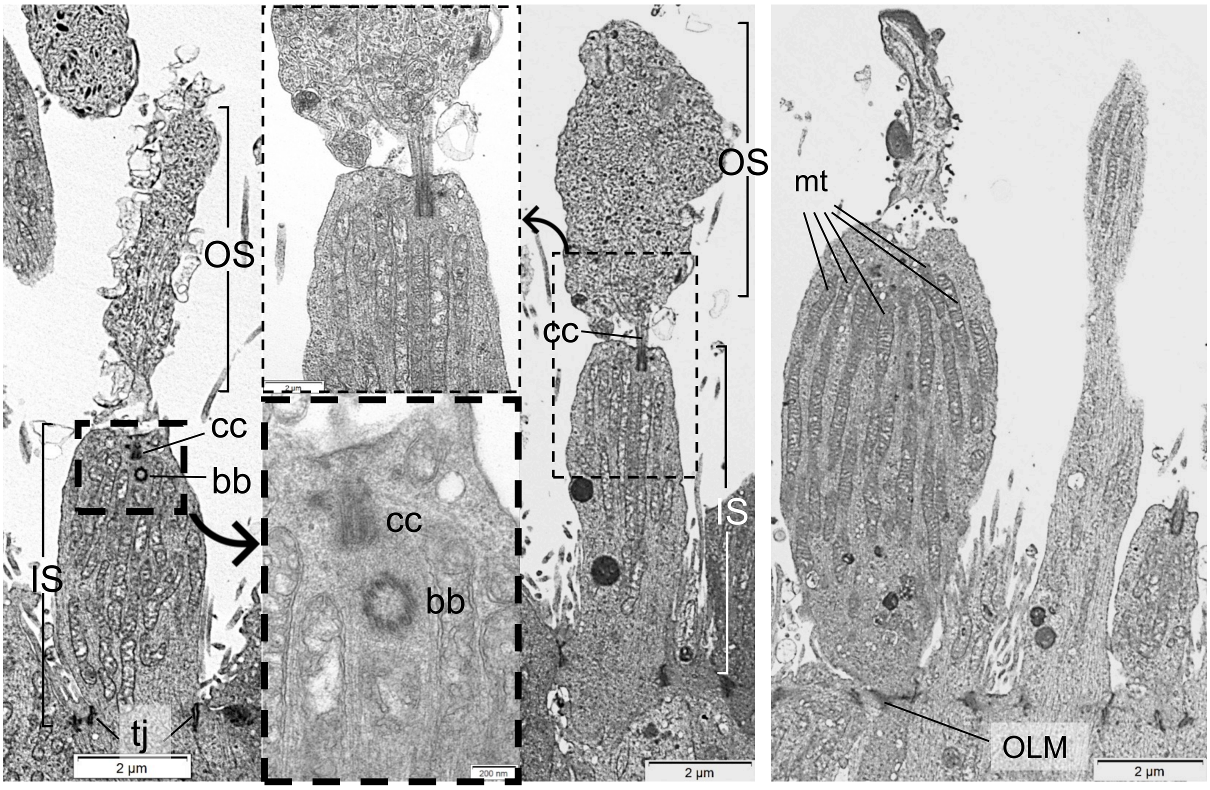

Figure S8

Supplementary Figure 9:

Examples of OVs cultured in different conditions (2D/3D AMM Xeno and xeno-free, XF) immunostained for beta-tubulin, TUBB3, which labels retinal ganglion cells and the plexiform layers shows variability of inner retinal lamination. ONL, presumptive outer nuclear layer. DAPI (blue) nuclear stain.

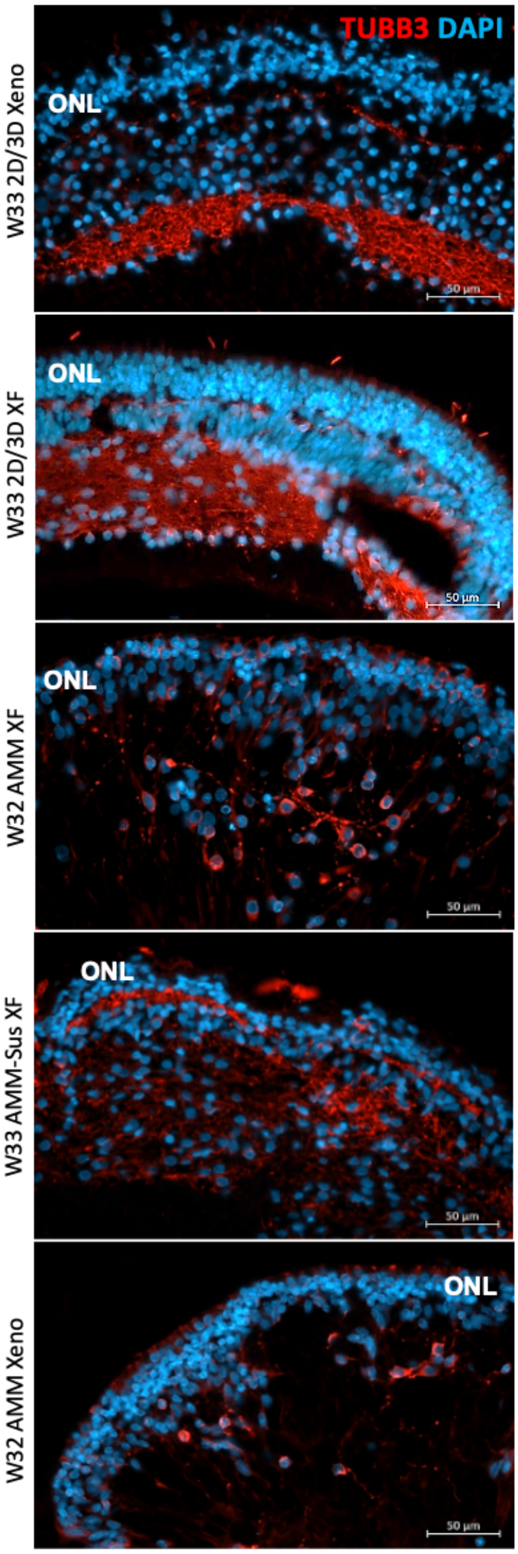
TUBB3 DAPI
W33 2D/3D Xeno
ONL
50 µm
W33 2D/3D XF
ONL
50 µm
W32 AMM XF
ONL
50 µm
W33 AMM-Sus XF
ONL
50 µm
W32 AMM Xeno
ONL
50 µm

Figure S9

Supplementary Data

Key resource table 1

**Primers**		
Gene Name	Catalogue Number	Source
GAPDH	Hs02758991_g1	Thermo Fisher Scientific
PAX6	Hs00240871_m1	Thermo Fisher Scientific
RAX	Hs00429459_m1	Thermo Fisher Scientific
VSX2	Hs01584047_m1	Thermo Fisher Scientific
CRX	Hs00230899_m1	Thermo Fisher Scientific
RCVRN	Hs00610056_m1	Thermo Fisher Scientific
ARR3	Hs00182888_m1	Thermo Fisher Scientific
NRL	Hs00172997_m1	Thermo Fisher Scientific
RHO	Hs00892431_m1	Thermo Fisher Scientific
OPN1SW	Hs00181790_m1	Thermo Fisher Scientific
OPN1LW	Hs00241039_m1	Thermo Fisher Scientific
MITF	Hs01117294_m1	Thermo Fisher Scientific
RPE65	Hs01071462_m1	Thermo Fisher Scientific
BEST	Hs00188249_m1	Thermo Fisher Scientific
GFAP	Hs009092331_m1	Thermo Fisher Scientific
**Primary Antibodies**		
Marker	Catalogue Number	Source
Mouse anti-CRX 1:400	H00001406-M02	Abnova
Rabbit anti-RCVRN 1:800	A85585	Merck Millipore
Rabbit anti-PAX6 1:200	901301	Biolegend
Goat anti-ARR3 1:200	NBP-37003	Novus
Rabbit anti-OTX1&2 1:200	Ab21990	Abcam
Mouse anti-NR2E3 1:400	PP-H7223-00	Persus Proteomics
Goat anti-NRL 1:500	AF2945	R&D Systems
Rabbit anti-Opsin S 1:200	AB5407	Merck Millipore
Rabbit anti-Opsin M/L 1:400	AB5405	Merck Millipore
Mouse anti-RHO 1:500	O4886	Sigma
Mouse anti-PKCα 1:50	GTX11723	GeneTex
Rabbit anti-ARL13B 1:50	17711-1-AP	Proteintech
Rabbit anti-GNAt1 (K-20) 1:50	SC-389	Santa Cruz Biotechnology
Mouse anti-PRPH2 1:200	MABN293	Merck Millipore
Mouse anti-TUBB3 1:200	T6793	Sigma
Mouse anti-SYN 1:100	AB8049	Abcam
Rabbit anti-ZO1 1:200	617300	Thermo Fisher Scientific
Mouse anti-BEST 1:200	MAB5466	Merck Millipore
**Secondary Antibodies**		
Marker	Catalogue Number	Source
Donkey Anti-mouse IgG 488 1:500	A21202	Invitrogen
Goat Anti-Rabbit IgG 568 1:500	A11063	Invitrogen
Goat Anti-Rabbit IgG 647 1:500	A21245	Invitrogen
Donkey Anti-Goat IgG 647 1:500	A21447	Invitrogen

Key resource table (continued)

**RIM1**			
Product Name	Catalogue Number	Source	Volume (ml)
G-MEM BHK-21	21710025	Thermo Fisher Scientific	384
Knockout Serum Replacement	10828028	Thermo Fisher Scientific	100
Gluta-MAX	35050038	Thermo Fisher Scientific	5
Na-Pyruvate	11360070	Thermo Fisher Scientific	5
2-Mercaptoethanol	31350010	Thermo Fisher Scientific	1
Penicillin-Streptomycin	15140122	Thermo Fisher Scientific	5
**RIM2-xeno**			
Product Name	Catalogue Number	Source	Volume (ml)
RIM1			450
FBS	16000044	Thermo Fisher Scientific	50
**RIM2-xeno free**			
Product Name	Catalogue Number	Source	Volume (ml)
RIM1			495
HPL	06960	Stemcell Technologies	5
**RIM3-xeno**			
Product Name	Catalogue Number	Source	Volume (ml)
DMEM: F12	10565018	Thermo Fisher Scientific	434.5
N2	17502001	Thermo Fisher Scientific	5
FBS	16000044	Thermo Fisher Scientific	50
100× MEM-NEAA	11140050	Thermo Fisher Scientific	5
Taurine	T4571-100G	Ajinamoto	0.5
Penicillin-Streptomycin	15140122	Thermo Fisher Scientific	5
**RIM3-xeno free**			
Product Name	Catalogue Number	Source	Volume (ml)
DMEM: F12	10565018	Thermo Fisher Scientific	479.5
N2	17502001	Thermo Fisher Scientific	5
HPL	06960	Stemcell Technologies	5
100× MEM-NEAA	11140050	Thermo Fisher Scientific	5
Taurine	T4571-100G	Ajinamoto	0.5
Penicillin-Streptomycin	15140-122	Thermo Fisher Scientific	5
**RMM-xeno**			
Product Name	Catalogue Number	Source	Volume (ml)
G-MEM BHK-21	21710-025	Thermo Fisher Scientific	429.5
B27	17502-001	Thermo Fisher Scientific	10
FBS	16000-044	Thermo Fisher Scientific	50
Na-Pyruvate	11360070	Thermo Fisher Scientific	5
Taurine	T4571-100G	Ajinamoto	0.5
Penicillin-Streptomycin	15140-122	Thermo Fisher Scientific	5
**RMM-xeno free**			
Product Name	Catalogue Number	Source	Volume (ml)
G-MEM BHK-21	21710-025	Thermo Fisher Scientific	474.5
B27	17502-001	Thermo Fisher Scientific	10
HPL	06960	StemCell Technologies	5
Na-Pyruvate	11360070	Thermo Fisher Scientific	5
Taurine	T4571-100G	Ajinamoto	0.5
Penicillin-Streptomycin	15140-122	Thermo Fisher Scientific	5

Key resource table (continued)

Small molecules

Product Name	Catalogue Number	Source
Y-27632 hydrochloride	10005583	Cayman Chemical Company
IWR1e	681669	Merck Millipore
SAG	566660	Merck Millipore
CHIR99021	361571	Merck Millipore
DAPT	D5942	Sigma
Retinoic Acid	R2625	Sigma

Extracellular Matrix

Product Name	Catalogue Number	Source
Matrigel Matrix	354277	Corning
Recombinant Human Laminin 521	LN521	BioLamina
